# Supplementary material for: Antisense Oligonucleotide-Based Therapeutic against Menin for Triple-Negative Breast Cancer Treatment
Source: Biomedicines. 2021 Jul 8;9(7):795. doi: 10.3390/biomedicines9070795 (PMC8301388; doi:10.3390/biomedicines9070795)
Supplement: Supplementary file 1 [file biomedicines-09-00795-s001.zip › biomedicines-1215247-supplementary-done.pdf]

biomedicines

MDPI

Supplementary

Antisense Oligonucleotide-Based Therapeutic against Menin for Triple-Negative Breast Cancer Treatment

Dang Tan Nguyen [1,†], Thi Khanh Le [1,2,†], Clement Paris [1,†], Chaïma Cherif [1], Stéphane Audebert [3], Sandra Oluchi Udu- Ituma [1], Sebastien Benizri [4], Philippe Barthelemy [4], François Bertucci [1], David Taïeb [1,5] and Palma Rocchi [1,*]

Table S1 List of potential menin-targeting ASOs utilized in the screening.

ASO Number	Position	Antisense 5'-3'	% GC
**6**	211-230	CACCAAGGAAAGGAGCACCA	55
**7**	231-250	AAATCGTCCACGAAGCCCAG	55
**8**	251-270	TGACGCGGTTGACAGCCAGA	60
**9**	271-290	CTCGGGAACGTTGGTAGGGA	60
**12**	331-350	CACGGGAAAGTAGGTGAGGC	60
**13**	351-370	GGATAGAGGGACAGGTCGAC	60
**16**	411-430	GGATAGAGGGACAGGTCGAC	60
**17**	431-450	TGGAGACACCCCCTTCTCGA	60
**18**	451-470	CTTCTTCACCAGCTCACGGC	60
**19**	471-490	TTCCATATGACATCGGAGAC	45
**21**	511-530	GATGTGGGCCCGATCCTTGA	60
**22**	531-550	ATGAAGCTGAAGAGGGACTG	50
**23**	551-570	TGTCCAATTTGGTGCCTGTG	50
**27**	631-650	ATCCTCAGACAGGGCGAGGT	60
**28**	651-670	CCAAACACTACCCAGGCATG	55
**34**	771-790	TATGATCCTTTCAGGTACAG	40
**35**	791-810	TCTTGCGGTCACAGCGCATG	60
**36**	811-830	CACCATGTTCGCCACCTCCA	50
**37**	831-850	TGATGGCACAATGGAAGGGT	50
**39**	871-890	CTGCAGCTGCAGAAGCTCCA	60
**40**	891-910	AGCAGCCAGAGCAGCTTCTG	60
**41**	911-930	CCAGATGTCCCAGGTCATAG	55
**43**	951-970	TCTAGATCTGCCAGGTTCCC	55
**46**	1011-1030	GCAATGCCCTTGTGGTAGAG	55
**48**	1051-1070	GGGGTAGATGTGTTCATCCC	55
**50**	1071-1090	CATTGCGGTTGCGACAGTGG	60
**63**	1351-1370	GATGCCGTCGTAGAATCGCA	55
**64**	1371-1390	CTGCCCTCCTCCCATTTGCA	60
**66**	1411-1430	AAGAAAGGTGGCCCAGCCCA	60
**67**	1431-1450	AAACGGCCTAGGGACTGCAC	60
**69**	1471-1490	TCGGCTCACTATGCGCACCT	60
**86**	1811-1830	CGCTCGAGTTGATCTTGGTG	55
**87**	1831-1850	CGTGAGTTGCAGCTTGATGG	55
**88**	1851-1870	ATCTGCACTTGCGACTGTGC	55
